# Updated efficacy of avelumab in patients with previously treated metastatic Merkel cell carcinoma after ≥1 year of follow-up: JAVELIN Merkel 200, a phase 2 clinical trial

**DOI:** 10.1186/s40425-017-0310-x

**Published:** 2018-01-19

**Authors:** Howard L. Kaufman, Jeffery S. Russell, Omid Hamid, Shailender Bhatia, Patrick Terheyden, Sandra P. D’Angelo, Kent C. Shih, Céleste Lebbé, Michele Milella, Isaac Brownell, Karl D. Lewis, Jochen H. Lorch, Anja von Heydebreck, Meliessa Hennessy, Paul Nghiem

**Affiliations:** 1 Rutgers Cancer Institute of New Jersey, 195 Little Albany Street, Room 2007, New Brunswick, NJ 08901 USA; 2 Present Address: Replimune Inc, Woburn, MA USA; 3 H. Lee Moffitt Cancer Center, Tampa, FL USA; 4 Present Address: Immunocore, Ltd, Conshohocken, PA USA; 5 The Angeles Clinic and Research Institute, Los Angeles, CA USA; 6 University of Washington Medical Center, Seattle, WA USA; 7 University of Lübeck, Lübeck, Germany; 8 Memorial Sloan Kettering Cancer Center & Weill Cornell Medical College, New York, NY USA; 9 Sarah Cannon Research Institute/Tennessee Oncology, Nashville, TN USA; 10 APHP Dermatology and CIC Departments, University Paris Diderot INSERM U976, Saint Louis Hospital, Paris, France; 11 Regina Elena National Cancer Institute, Rome, Italy; 12 National Cancer Institute, Bethesda, MD USA; 13 University of Colorado Denver, School of Medicine, Aurora, CO USA; 14 Dana-Farber Cancer Institute, Boston, MA USA; 15 Merck KGaA, Darmstadt, Germany; 16 EMD Serono, Inc, Billerica, MA USA; 17 University of Washington Medical Center at South Lake Union, Seattle, WA USA

**Keywords:** Javelin, Avelumab, Merkel cell carcinoma, Pd-L1

## Abstract

**Background:**

Merkel cell carcinoma (MCC) is a rare, aggressive skin cancer associated with poor survival outcomes in patients with distant metastatic disease (mMCC). In an initial analysis from JAVELIN Merkel 200, a phase 2, prospective, open-label, single-arm trial in mMCC, avelumab—a human anti–programmed death-ligand 1 (PD-L1) monoclonal antibody—showed promising efficacy and a safety profile that was generally manageable and tolerable. Here, we report the efficacy of avelumab after ≥1 year of follow-up in patients with distant mMCC that had progressed following prior chemotherapy for metastatic disease.

**Patients and methods:**

Patients received avelumab 10 mg/kg by 1-h intravenous infusion every 2 weeks until confirmed disease progression, unacceptable toxicity, or withdrawal. The primary endpoint was best overall response. Secondary endpoints included duration of response (DOR), progression-free survival (PFS), and overall survival (OS).

**Results:**

Patients (*N* = 88) were followed for a minimum of 12 months. The confirmed objective response rate was 33.0% (95% CI, 23.3%-43.8%; complete response: 11.4%). An estimated 74% of responses lasted ≥1 year, and 72.4% of responses were ongoing at data cutoff. Responses were durable, with the median DOR not yet reached (95% CI, 18.0 months-not estimable), and PFS was prolonged; 1-year PFS and OS rates were 30% (95% CI, 21%-41%) and 52% (95% CI, 41%-62%), respectively. Median OS was 12.9 months (95% CI, 7.5-not estimable). Subgroup analyses suggested a higher probability of response in patients receiving fewer prior lines of systemic therapy, with a lower baseline disease burden, and with PD-L1–positive tumors; however, durable responses occurred irrespective of baseline factors, including tumor Merkel cell polyomavirus status.

**Conclusions:**

With longer follow-up, avelumab continues to show durable responses and promising survival outcomes in patients with distant mMCC whose disease had progressed after chemotherapy.

**Trial registration:**

Clinicaltrials.gov identifier: NCT02155647.

## Introduction

Merkel cell carcinoma (MCC) is a rare, aggressive skin cancer associated with clonal integration of Merkel cell polyomavirus (MCPyV), accumulation of UV-induced DNA mutations, immunosuppression, and old age [1, 2]. Up to 12% of patients with MCC have distant metastatic disease (mMCC), which has a poor prognosis [1, 3], and progression to mMCC is frequent in patients with local or regional disease (up to 21%) [4]. Although no prospective clinical trials of chemotherapy have been conducted and no regimen has been specifically approved for mMCC treatment, platinum/etoposide combinations have been widely used and achieve relatively high objective response rates (ORRs); response duration, however, is short and no clear survival advantage has been reported [5, 6], highlighting the need for alternative treatments. Recently, clinical trials with immune checkpoint inhibitors targeting the programmed death-ligand 1 (PD-L1)/programmed death 1 (PD-1) interaction have shown clinical activity and durable responses in patients with advanced MCC [7–9]. Based on findings from an open-label, single-arm, prospective, phase 2 trial [8], avelumab—a human anti–PD-L1 monoclonal antibody—became the first treatment approved by the US Food and Drug Administration (FDA) for patients with mMCC [10]. Here, we report updated efficacy data for avelumab with ≥1 year of follow-up in patients with mMCC that had progressed after ≥1 prior line of chemotherapy for metastatic disease.

## Methods

### Study design and patients

The procedures for analysis and design of the JAVELIN Merkel 200 trial (NCT02155647) were reported previously [8]. Briefly, patients with histologically confirmed stage IV MCC that had progressed following ≥1 prior line of chemotherapy for metastatic disease, were enrolled. Eligible patients were adults aged ≥18 years who had Eastern Cooperative Oncology Group (ECOG) performance status of 0 or 1, an estimated life expectancy of ≥3 months, ≥1 unidimensional measurable lesion by Response Evaluation Criteria In Solid Tumors (RECIST) version 1.1 [11], and adequate hematological, hepatic, and renal function. Patients who received previous therapy with immune checkpoint inhibitor or concurrent anticancer treatment, systemic treatment with corticosteroids, or those with HIV, immunosuppression, previous organ transplant, hematological malignancies, or clinically significant comorbidities were excluded. Patients were not selected based on tumor PD-L1 expression or MCPyV status. Patients received avelumab 10 mg/kg by 1-h intravenous infusion every 2 weeks until confirmed disease progression, unacceptable toxicity, or occurrence of any other criterion for withdrawal.

### Outcomes and statistical analysis

The primary endpoint was best overall response—defined as complete response (CR), partial response (PR), stable disease, or progressive disease per RECIST v1.1—and was evaluated by an independent review committee every 6 weeks. Secondary endpoints included duration of response (DOR), progression-free survival (PFS), and overall survival (OS), and a post hoc analysis was carried out to determine the 6-month durable response rate (DRR) [8]. Time-to-event endpoints were analyzed by Kaplan-Meier methods; medians were calculated with corresponding CIs using the Brookmeyer-Crowley method. Safety data are summarized in aggregate for this report and are reported elsewhere [10, 12].

## Results

Baseline characteristics of the 88 patients enrolled and treated with avelumab were reported previously [8]. Briefly, median age was 72.5 years (range, 64.5-77.0), 65 patients (74%) were male, 47 patients (53.4%) had visceral disease at baseline (ie, any lesions identified in sites other than skin, eye, or lymph nodes by independent review), 49 (55.7%) and 39 (44.3%) had an ECOG performance status score of 0 or 1, and 52 (59.1%), 26 (29.5%), and 10 patients (11.4%) had received 1, 2, or ≥3 prior lines of anticancer treatment, respectively.

**Fig. 1:**
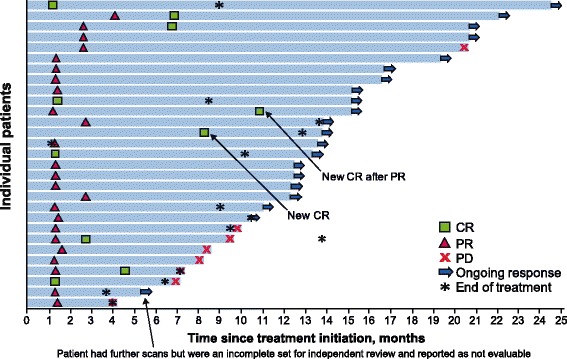
Clinical activity of avelumab in patients with mMCC at ≥1 year of follow-up. Time to and duration of response and duration of treatment in 29 patients with a confirmed response. CR, complete response; DOR, duration of response; PD, progressive disease; PR, partial response

As of September 3, 2016 (data cutoff date), median follow-up was 16.4 months (range, 12.1-25.4). Treatment was ongoing in 19 patients (21.6%), and 69 patients (78.4%) had discontinued treatment—mostly due to disease progression (*n* = 44 [63.8%]) or adverse events (*n* = 7 [10.1%]), which were treatment-related in 6 patients and included ileus and transaminitis. Ten patients (11.4%) had a confirmed CR, including 2 new CRs since the primary analysis [8], and 19 patients (21.6%) had a PR, resulting in an ORR of 33.0% (95% CI, 23.3%-43.8%) (Table 1). Median time to response was 6.1 weeks (range, 6-36), with 22 of 29 responses (75.9%) observed 6 weeks after treatment initiation. Responses were ongoing at data cutoff in 21 of 29 patients (72.4%), including in 9 patients (31.0%) who had reached the end of treatment (Fig. 1). Responses were durable, with the median DOR not yet reached; the lower bound for the 95% CI was 18.0 months, and the longest observed DOR was 23.3 months in a patient with ongoing response (Table 1, Fig. 1). The estimated proportion of responses with a duration ≥1 year was 74% (95% CI, 53%-87%). The 6-month DRR was 30.6% (95% CI, 20.9%-40.3%), and the overall proportion of patients in response at 1 year after treatment initiation was 23.9% (95% CI, 15.4%-34.1%).

**Table 1:** Efficacy of avelumab after ≥6 months and ≥1 year of follow-up

Efficacy parameter	≥6 months of follow-up [8]	≥1 year of follow-up
	(*N* = 88)	(*N* = 88)
ORR (95% CI), %	31.8 (21.9-43.1)^a^	33.0 (23.3-43.8)
Confirmed BOR, n (%)		
CR	8 (9.1)	10 (11.4)
PR	20 (22.7)	19 (21.6)
SD	9 (10.2)	9 (10.2)
PD	32 (36.4)	32 (36.4)
Non-CR/non-PD	1 (1.1)^b^	0
Not evaluable^c^	18 (20.5)	18 (20.5)
Response durability	(*n* = 28)	(*n* = 29)
Median DOR (95% CI), months	NE (8.3-NE)	NE (18.0-NE)
Range	2.8-17.5+	2.8-23.3+
6-month DRR (95% CI), %^d^	29.1 (19.5-38.8)	30.6 (20.9-40.3)
Proportion of responses with duration ≥6 months (95% CI), %^e^	92 (70-98)	93 (74-98)
Proportion of responses with duration ≥1 year (95% CI), %^e^	NA	74 (53-87)
Proportion of patients in response at 1 year (95% CI), %^f^	NA	23.9 (15.4-34.1)

*BOR* Best overall response, *CR* Complete response, *DOR* Duration of response, *DRR* Durable response rate, *NA* Not applicable, *NE* Not estimable, *ORR* Objective response rate, *PD* Progressive disease, *PR* Partial response, *SD* Stable disease

^a^ 95.9% CI adjusted for multiple testing

^b^ One patient did not have measurable disease at baseline; thus, a BOR of PR or SD could not be distinguished

^c^ Patients not evaluable for a confirmed BOR had no baseline lesions identified by independent review committee (*n* = 4), baseline but no postbaseline assessments (*n* = 10), all nonassessable postbaseline assessments (*n* = 2), no postbaseline tumor assessment before the start of new anticancer therapy (*n* = 1), or SD of insufficient duration (*n* = 1)

^d^ ORR multiplied by Kaplan-Meier estimate for proportion of responses with a duration of ≥6 months

^e^ Based on Kaplan-Meier estimates

^f^ 95% exact CI using the Clopper-Pearson method

**Fig. 2:**
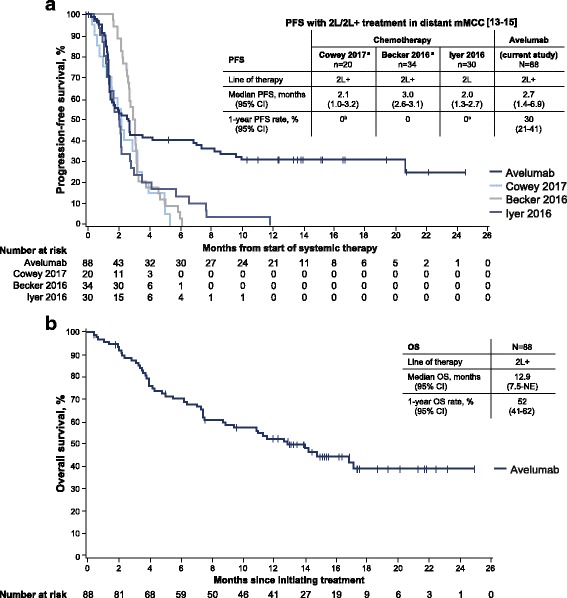
Survival outcomes in patients with mMCC receiving avelumab. Kaplan-Meier estimates of (**a**) progression-free survival (PFS) and (**b**) overall survival (OS). Vertical lines indicate censored events. Also depicted in (**a**) are Kaplan-Meier estimates of PFS for recent retrospective studies of second-line (2 L) or second-line and later (2 L+) chemotherapy in patients with mMCC [13–15]. NE, not estimable. ^a^ Includes both immunocompetent and immunocompromised patients. All patients progressed; therefore, none were censored. ^b^ PFS rate at 6 months was 0%. ^c^ One patient with PR had PFS lasting 354 days; 95% of patients receiving second-line chemotherapy had progressed at 230 days

The 1-year PFS rate was 30% (95% CI, 21%-41%), and median PFS was 2.7 months (95% CI, 1.4-6.9); the maximum time reported at cutoff was 24.5 months (Fig. 2a). For illustrative purposes, Kaplan-Meier estimates of PFS from recent studies of second-line or later chemotherapy for mMCC are also depicted [13–15]. Median OS was 12.9 months (95% CI, 7.5-not estimable), and the 1-year OS rate was 52% (95% CI, 41%-62%) (Fig. 2b).

**Fig. 3:**
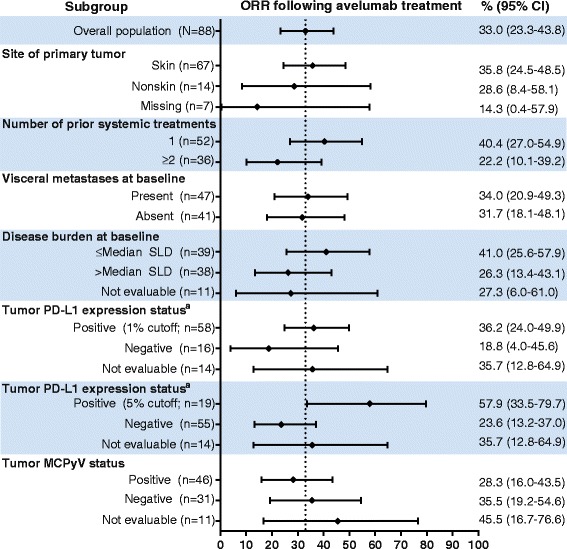
Objective response rates in patient subgroups. The ORR and associated 95% CI values are graphed and shown for the indicated subgroups. MCPyV, Merkel cell polyomavirus; ORR, objective response rate; PD-L1, programmed death-ligand 1; SLD, sum of target lesion diameters. ^a^ PD-L1 expression in tumor samples was assessed using a proprietary immunohistochemistry assay (Dako PD-L1 IHC 73-10 pharmDx). Determination of PD-L1–positive status at different PD-L1 cutoff levels was based on tumor cell staining of any intensity

**Fig. 4:**
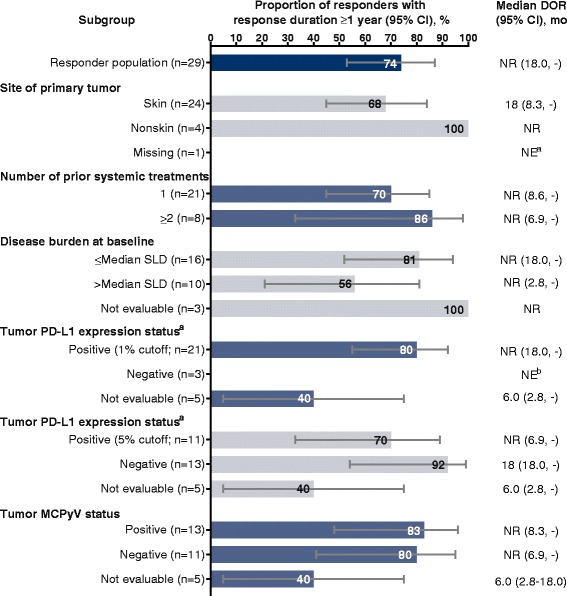
Response durability in patient subgroups. The proportions of responding patients with response duration ≥1 year are depicted for the indicated patient subgroups. The associated median DOR and 95% CI for each subgroup is shown on the right. DOR, duration of response; MCPyV, Merkel cell polyomavirus; NE, not estimable; NR, not yet reached; PD-L1, programmed death-ligand 1; SLD, sum of target lesion diameters. ^a^ One patient missing information on site of the primary tumor had an ongoing response for <1 year (8.8+ months). ^b^ Of 3 patients with a response to avelumab and PD-L1–negative status (<1% tumor-cell staining cutoff), the response was ongoing in all 3 patients for <1 year (3.9+, 11.1+, and 11.1+ months)

Subgroup analyses showed trends for higher ORR in patients who received fewer prior lines of anticancer treatment (1 vs ≥2 prior lines, 40.4% vs 22.2%), with lower disease burden (sum of target lesion diameters ≤ median vs > median, 41.0% vs 26.3%), and with PD-L1–positive tumors (1% threshold by immunohistochemistry, 36.2% vs 18.8% for PD-L1–negative tumors; 5% threshold by immunohistochemistry, 57.9% vs 23.6% for PD-L1–negative tumors) (Fig. 3). The proportions of responses with ≥1-year duration were similar across evaluable subgroups, including tumor MCPyV status (Fig. 4).

## Discussion

These updated data from 88 patients with distant mMCC that progressed following ≥1 line of prior chemotherapy show that avelumab treatment resulted in durable efficacy and prolonged PFS. The confirmed ORR was 33.0%, which is higher than ORRs reported in recent observational studies of second-line or later chemotherapy (9%-23%) [13–15]. Responses were durable, as evidenced by most (72.4%) ongoing at data cutoff and a median DOR not yet reached; the lower bound of the 95% CI (18.0 months) was considerably longer than the median DOR in retrospective studies of chemotherapy (1.7-3.3 months) [13–15]. Furthermore, the estimated proportion of responses lasting ≥1 year with avelumab was 74%, whereas few patients in the historical chemotherapy reference literature had a response lasting 6 months 13-15]. Kaplan-Meier plots of PFS showed that a notable proportion of avelumab-treated patients, primarily those with response, have ongoing clinical benefit. Although median PFS was similar to that with chemotherapy, the Kaplan-Meier–estimated PFS curve for avelumab reached a plateau, which is unprecedented with chemotherapy. Furthermore, the 1-year rate of PFS was 30% in avelumab-treated patients compared with 0% in chemotherapy-treated patients (Fig. 2a). Median OS was 12.9 months, compared with values of <6 months with second-line or later chemotherapy in patients with mMCC [13–15], and the lower bound of the 95% CI for median OS (7.5 months) was longer than that reported in these retrospective studies (Fig. 2b). As for PFS, the Kaplan-Meier estimated OS curve also reached a plateau; approximately 40% of patients exhibited long-term survival. These promising data underscore the challenges to conducting a randomized phase 3 study of immunotherapy compared with chemotherapy in this patient population, given the clear survival benefit of immunotherapy.

We observed objective responses across all subgroups and noted a trend for higher ORRs in patients who received fewer lines of prior therapy, who had lower disease burden, and whose tumors were PD-L1–positive (Fig. 3); these patients might be more likely to be immunocompetent and thus more responsive to immune checkpoint inhibitor treatment [16]. Durable responses were seen across all patient subgroups, irrespective of tumor PD-L1 and MCPyV status (Fig. 4). Taken together, these findings suggest that avelumab may be clinically active in patients with mMCC with different mechanisms of oncogenesis.

JAVELIN Merkel 200 is the largest prospective clinical trial performed in mMCC to date and is continuing enrollment of an additional cohort of patients with mMCC who will receive avelumab as first-line treatment. Findings from this study led to accelerated FDA approval of avelumab for the treatment of patients with mMCC [8, 10], and, for the first time, offer an alternative to chemotherapy.

## Conclusions

Avelumab monotherapy has durable antitumor activity in patients with mMCC that progressed after chemotherapy. With a minimum of 1 year of follow-up, the observed prolonged PFS and maturing OS data suggest a potential long-term benefit not previously reported with chemotherapy.

## Abbreviations

2 L: Second-line
2 L +: Second-line or later
CI: Confidence interval
CR: Complete response
DOR: Duration of response
DRR: Durable response rate
ECOG: Eastern Cooperative Oncology Group
FDA: Food and Drug Administration
HIV: Human immunodeficiency virus
MCPyV: Merkel cell polyomavirus
MMCC: Metastatic Merkel cell carcinoma
NE: Not estimable
NR: Not yet reached
ORR: Objective response rate
OS: Overall survival
PD: Progressive disease
PD-1: Programmed death-1
PD-L1: Programmed death-ligand 1
PFS: Progression-free survival
PR: Partial response
RECIST: Response Evaluation Criteria In Solid Tumors
SLD: Sum of target lesion diameters
UV: Ultraviolet

## References

[1] Lebbe C, Becker JC, Grob JJ, Malvehy J, Del Marmol V, Pehamberger H (2015). Diagnosis and treatment of Merkel cell carcinoma. European consensus-based interdisciplinary guideline. Eur J Cancer.

[2] Terheyden P, Becker JC. New developments in the biology and the treatment of metastatic Merkel cell carcinoma. Curr Opin Oncol. 2017; March 9. [Epub ahead of print] 10.1097/CCO.0000000000000363 28282342

[3] Schadendorf D, Lebbe C, Zur Hausen A, Avril MF, Hariharan S, Bharmal M (2017). Merkel cell carcinoma: epidemiology, prognosis, therapy and unmet medical needs. Eur J Cancer.

[4] Allen PJ, Bowne WB, Jaques DP, Brennan MF, Busam K, Coit DG (2005). Merkel cell carcinoma: prognosis and treatment of patients from a single institution. J Clin Oncol.

[5] NCCN (2018). Clinical practice guidelines in oncology: Merkel cell carcinoma.

[6] Nghiem P, Kaufman HL, Bharmal M, Mahnke L, Phatak H, Becker JC (2017). Systematic literature review of efficacy, safety and tolerability outcomes of chemotherapy regimens in patients with metastatic Merkel cell carcinoma. Future Oncol.

[7] Nghiem PT, Bhatia S, Lipson EJ, Kudchadkar RR, Miller NJ, Annamalai L (2016). PD-1 blockade with pembrolizumab in advanced Merkel cell carcinoma. N Engl J Med.

[8] Kaufman HL, Russell J, Hamid O, Bhatia S, Terheyden P, D'Angelo SP (2016). Avelumab in patients with chemotherapy-refractory metastatic Merkel cell carcinoma: a multicentre, single-group, open-label, phase 2 trial. Lancet Oncol.

[9] Topalian SL, Bhatia S, Hollebecque A, Awada A, De Boer JP, Kudchadkar RR, et al. Non-comparative, open-label, multiple cohort, phase 1/2 study to evaluate nivolumab (NIVO) in patients with virus-associated tumors (CheckMate 358): efficacy and safety in Merkel cell carcinoma (MCC). Cancer Res 2017;77(Suppl 13 [Abstract CT074]). doi:10.1158/1538-7445.AM2017-CT074.

[10] Bavencio (2017). (avelumab) injection [package insert].

[11] Eisenhauer EA, Therasse P, Bogaerts J, Schwartz LH, Sargent D, Ford R (2009). New response evaluation criteria in solid tumours: revised RECIST guideline (version 1.1). Eur J Cancer.

[12] Kaufman HL, Russell JS, Hamid O, Bhatia S, Terheyden P, D'Angelo SP, et al. Durable responses to avelumab (anti–PD-L1) in patients with Merkel cell carcinoma progressed after chemotherapy: 1-year efficacy update. Cancer Res 2017;77(Suppl 13 [Abstract CT079]). doi:10.1158/1538-7445.AM2017-CT074.

[13] Cowey CL, Mahnke L, Espirito J, Helwig C, Oksen D, Bharmal M (2017). Real-world outcomes of patients with metastatic Merkel cell caricnoma treated with chemotherapy in the USA. Future Oncol.

[14] Becker J, Lorenz E, Haas G, Helwig C, Oksen D, Mahnke L, et al. Evaluation of real world treatment outcomes in patients with metastatic Merkel cell carcinoma (MCC) following second line chemotherapy. Ann Oncol 2016;26(Suppl 3 [Abstract 2602]). doi:10.1158/1538-7445.AM2017-CT074.

[15] Iyer JG, Blom A, Doumani R, Lewis C, Tarabadkar ES, Anderson A (2016). Response rates and durability of chemotherapy among 62 patients with metastatic Merkel cell carcinoma. Cancer Med.

[16] Schadendorf D, Nghiem P, Bhatia S, Hauschild A, Saiag P, Mahnke L, et al. Immune evasion mechanisms and immune checkpoint inhibition in advanced Merkel cell carcinoma. OncoImmunology. 2017; 10.1080/2162402X.2017.1338237. 10.1080/2162402X.2017.1338237 PMC5665072 29123950

